# Supplementary material for: Kin but less than kind: within-group male relatedness does not increase female fitness in seed beetles
Source: Proc Biol Sci. 2019 Sep 11;286(1910):20191664. doi: 10.1098/rspb.2019.1664 (PMC6742989; doi:10.1098/rspb.2019.1664)
Supplement: Supplementary figure 1 [file rspb20191664supp1.docx]

Supplementary material

**Kin but less than kind: within-group male relatedness does not increase female fitness in seed beetles**

Elena C. Berg, Martin I. Lind, Shannon Monahan,
Sophie Bricout and Alexei A. Maklakov

**Supplementary figure 1.** Lifetime reproductive success LRS (number of adult offspring) by treatment group, when including females that did not reproduce: brothers (blue), non-related males (red), individuals raised in group (solid symbols) or alone (open symbols). Top panel shows raw data, with the mean ± 95%CI indicated with black bars at each group. Bottom panel shows an estimation plot, where treatment levels are compared to NG, with a graded sampling distribution of bootstrapped values with a 95% CI.
